# Supplementary material for: Characterization of BRCA Deficiency in Ovarian Cancer
Source: Cancers (Basel). 2023 Feb 28;15(5):1530. doi: 10.3390/cancers15051530 (PMC10001116; doi:10.3390/cancers15051530)
Supplement: Supplementary file 1 [file cancers-15-01530-s001.zip › cancers-2221990-supplementary.pdf]

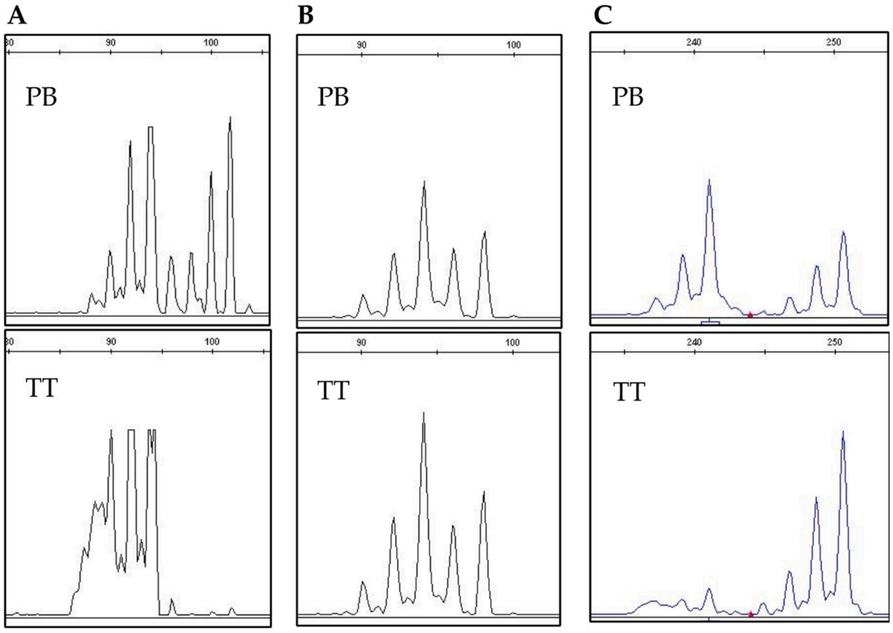


**Figure S1.** Microsatellite analysis in OC tissues.

A: In GECO 23, where MLPA had detected BRCA2 duplication, the analysis of D13S153 marker is consistent with the duplication, but also shows LOH, which extends to the entire chromosome, according to the status of the other markers analysed (not shown).

B: In GECO 30, also found to harbor BRCA2 duplication, microsatellite marker D13S153 analysis rules out LOH.

C: In GECO 34, the analysis of microsatellite marker D17S938 demonstrates LOH at BRCA1 region, despite no CNV detected by MLPA (“Copy-neutral” LOH: CN-LOH).

PB = Peripheral blood; TT = SF tumor tissue; LOH= Loss Of Heterozygosity

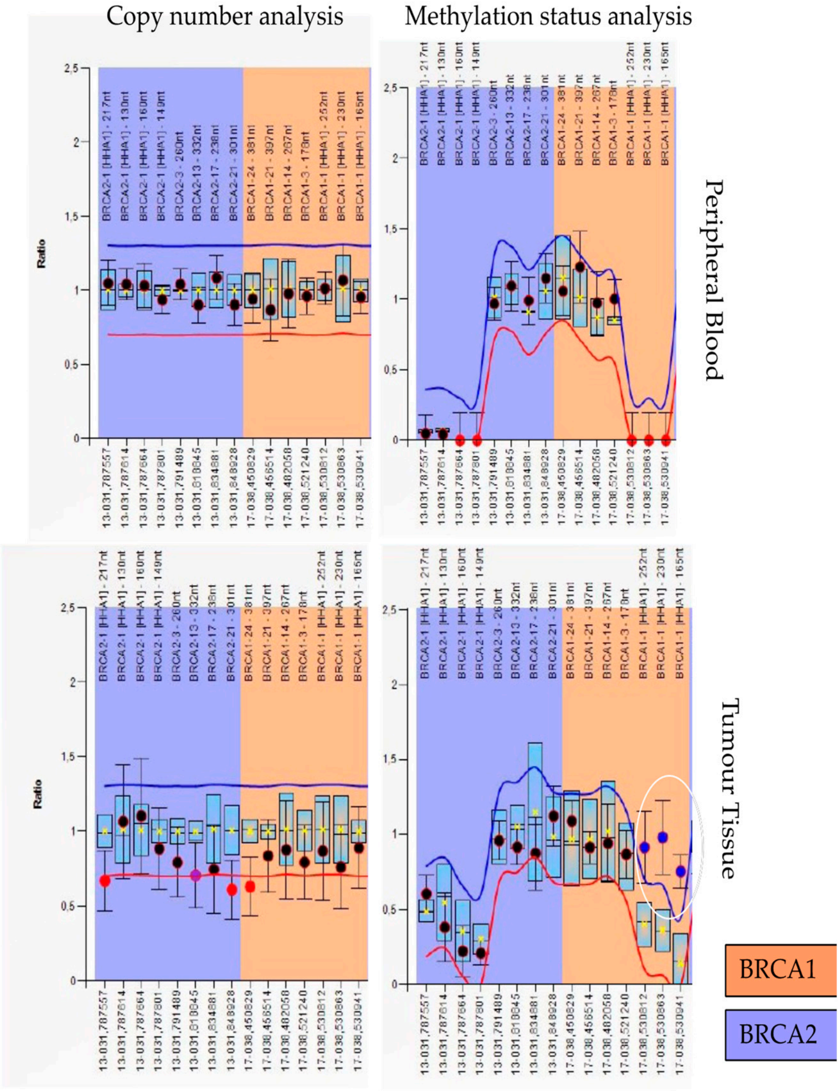


**Figure S2**. Promoter methylation plots in GECO 34.

**Table S1. Comprehensive results of the molecular analyses performed on OC tissues in this study.**

| Patient | BRCA status | *TP53* variant (classification[a]) | *TP53* variant load (%) | Other gene variants (classification[a]) | Other gene variant load (%) | Number of CNVs detected by SNP-CGH | Germline variants[b] (gene) |
|---|---|---|---|---|---|---|---|
| GECO 1 | BU | c.584T>C;p.Ile195Thr (P) | 72.1 | *ATM*: c.1272T>C;p.Pro424= (CI) | 42.7 | n.a. | / |
| GECO 2 | BU | N | / | *ATM*: c.2978A>Gp.His993Arg (US) | 47.3 | n.a. | / |
| GECO 3 | BD | n.a. | / | n.a. | / | n.a. | / |
| GECO 5 | BD | n.a. | / | n.a. | / | n.a. | / |
| GECO 6 | BU | c.395A>G;p.Lys132Arg (US) | 59.7 | N | / | 99 | / |
| GECO 7 | BD | n.a. | / | n.a. | / | 205 | / |
| GECO 8 | BD | n.a. | / | n.a. | / | 78 | *BRCA1*: c.5123C>A; p.Ala1708Glu |
| GECO 9 | BU | N | / | *ATM*: c.4060C>A;p.Pro1354Thr (CI) | 58.9 | 48 | / |
| GECO 12 | BU | c.990_991insT;p.Gln331SerfsTer6 (U) | 76.2 | N | / | n.a. | / |
| GECO 13 | BD | n.a. | / | n.a. | / | 89 | *BRCA2*: c.7558C>T; p.Arg2520Ter |
| GECO 14 | BU | c.818G>A;p.Arg273His (P/LP) | 63.8 | *RAD51C*: c.904+5G>T;p.? (LP) | 79.4 | n.a. | *RAD51C*: c.904+5G>T;p.? |
| GECO 15 | BU | c.380C>T;p.Ser127Phe (CI) | 54.5 | *PALB2*: c.1001A>G;p.Tyr334Cys (US) | 60.9 | n.a. | / |
| GECO 16 | BU | c.730G>A;p.Gly244Ser (P/LP) | 59.2 | *ATM*: c.2932T>C;p.Ser978Pro (CI) | 89.9 | 49 | / |

| | | | | | | | |
|---|---|---|---|---|---|---|---|
| GECO 17 | BU | c.649delG;p.Val217TrpfsTer30 (P) | 80.0 | *ATM*: c.7475T>G;p.Leu2492Arg (US) | 76.7 | n.a. | / |
| GECO 18 | BU | N | / | N | / | 15 | / |
| GECO 20 | BD | n.a. | / | n.a. | / | n.a. | / |
| GECO 21 | BU | c.469delG;p.Val157SerfsTer13 (U) | 66.1 | N | / | n.a. | / |
| GECO 22 | BU | c.661G>T;p.Glu221Ter (U) | 75.6 | *PTEN*: c.388C>T;p.Arg130Ter (P) | 56.5 | n.a. | / |
| GECO 23 | BU | c.818G>A;p.Arg273His (P/LP) | 93.9 | No | / | n.a. | / |
| GECO 24 | BD | n.a. | / | n.a. | / | n.a. | *BRCA2*: c.1813delA; p.Gly602=fs*11 |
| GECO 25 | BU | N | / | N | / | 107 | / |
| GECO 26 | BD | n.a. | / | n.a. | / | 196 | / |
| GECO 27 | BD | n.a. | / | n.a. | / | n.a. | *BRCA2*: c.9118-1G>A;p.? |
| GECO 28 | BU | N | / | N | / | n.a. | / |
| GECO 29 | BD | n.a. | / | n.a. | / | 72 | *BRCA1*: c.4065_4068delTCAA; p.Asn1355Lysfs |
| GECO 30 | BU | c.818G>T;p.Arg273Leu (P) | 65.6 | No | / | 37 | / |
| GECO 31 | BD | n.a. | / | n.a. | / | 90 | *BRCA1*: c.547+2T>A;p.? |
| GECO 33 | BU | c.742C>T;p.Arg248Trp (P) | 50.0 | N | / | n.a. | / |
| GECO 34 | BD | n.a. | / | n.a. | / | n.a. | / |
| GECO 35 | BU | c.824G>A;p.Cys275Tyr (P/LP) | 85.6 | *APC*: c.607C>G;p.Gln203Glu (CI) | 91.0 | 60 | / |

[a]ClinVar (P = Pathogenic; US = Uncertain Significance; LP = Likely Pathogenic; CI = Conflicting Interpretations; U = Unreported)

[b]Pathogenic or Likely Pathogenic

N = No; n.a. = not applicable (not performed)
